# Coronary Artery Disease and Gallbladder Inflammatory Pseudopolyps

**DOI:** 10.3390/diagnostics12010155

**Published:** 2022-01-10

**Authors:** Margherita Fosio, Giulia Cherobin, Roberto Stramare, Matteo Fassan, Chiara Giraudo

**Affiliations:** 1 UOSD Imaging Avanzato Clinico e Translazionale, Department of Medicine, University of Padova, 35127 Padova, Italy; margherita.fosio@gmail.com (M.F.); giuliachero@gmail.com (G.C.); roberto.stramare@unipd.it (R.S.); 2 Surgical Pathology, Department of Medicine, University of Padova, 35121 Padova, Italy; matteo.fassan@unipd.it; 3 Veneto Institute of Oncology, IOV-IRCCS, 35128 Padova, Italy

**Keywords:** gallbladder inflammatory polyps, MR, pseudotumor

## Abstract

Axial MR image demonstrating multiple small gallbladder polypoid lesions characterized by contrast enhancement in a 78-year-old male hospitalized for acute chest pain due to coronary artery disease who showed fever and emesis during hospitalization and had signs of acute acalculous cholecystitis at computed tomography. Given the overall clinical conditions and the MR features, the inflammatory origin of the polyps was considered. The patient underwent cholecystectomy and the histological diagnosis of gallbladder inflammatory pseudopolyps was confirmed. This rare entity represents 5–10% of all gallbladder polyps, and their differentiation from benign and malignant tumors might be challenging especially in acalculous patients, thus surgery is often performed.

A 78-year-old man was admitted to the emergency room of our tertiary center for acute chest pain due to coronary artery disease and underwent coronary artery bypass. During the hospitalization he had fever and emesis. Thus, he underwent contrast enhanced computed tomography demonstrating acute acalculous cholecystitis with localized gallbladder perforation (Figure 1a). Ultrasound-guided percutaneous transhepatic cholecystostomy was performed and antibiotic therapy administered. Ninety days later, a Magnetic Resonance (MR) scan showed multiple small gallbladder polyps characterized by contrast enhancement without any evidence of gallbladder stones (Figure 1b). The radiological findings were compatible with an inflammatory etiology of the polypoid lesions although, especially because of the lack of gallbladder stones and previous MR scans, neoplastic polyps could not be completely excluded. The patient underwent cholecystectomy and the histological diagnosis of gallbladder inflammatory pseudopolyps (GIP) was confirmed (Figure 2a,b).

Acute acalculous cholecystitis, even complicated by perforation, often occurs in post-surgical adults while in children it is often caused by infectious disease or immune-mediated disorders. Moreover, it can be associated with cardiovascular diseases, in particular with coronary artery disease and in children with Kawasaki Disease [1,2,3,4]. The etiology of acalculous cholecystitis is often unknown, even though it has been correlated with biliary hypokinesia and local ischemia [5,6,7,8].

The radiological literature regarding rare pseudotumors like GIP, which represents 5–10% of all gallbladder polyps, is scarce, and, even if they are usually multiple and small (<10 mm), a differentiation from benign and malignant tumors might be challenging. Mucosal irritation, granulation, and fibrous tissue, usually due to gallstones and/or chronic inflammation may cause GIPs [9,10,11].

The rarity of our case in which the occurrence of GIP is probably related to acalculous cholecystitis subsequent to acute coronary artery disease, is highlighted by the results of the brief literature search, without any restrictions on language and publication date, we conducted on Pubmed on the 4 January 2022. Indeed, using the keywords “(gallbladder inflammatory pseudopolyps) AND (coronary artery disease)” no records could be identified while applying the keywords “(acute acalculous cholecystitis) AND (coronary artery disease)” and “(acalculous cholecystitis) AND (inflammatory polyps)”, only 16 and one records were identified, respectively [12,13,14,15,16,17,18,19,20,21,22,23,24,25,26,27,28]. Three of the 16 records were excluded because they were not fully matching [25,26,27] and the single record deriving from the second search is not reported in Table 1 since it is a narrative review [28]. The records of the search are summarized in Table 1.

Thus, our case demonstrates that, although rarely, GIP may occur in patients with acalculous cholecystitis even associated with coronary artery disease and can be easily diagnosed at MR. Given the clinical course of our patient, the etiology and the benign nature of the pseudopolyps could have been assumed. Nevertheless, since the malignant behavior of gallbladder lesions is not easily excluded at imaging, especially in acalculous and symptomatic patients with an unknown/partially known clinical history, surgery still plays a dominant role and histology remains the gold standard for a precise characterization.

## Figures and Tables

**Figure 1:**
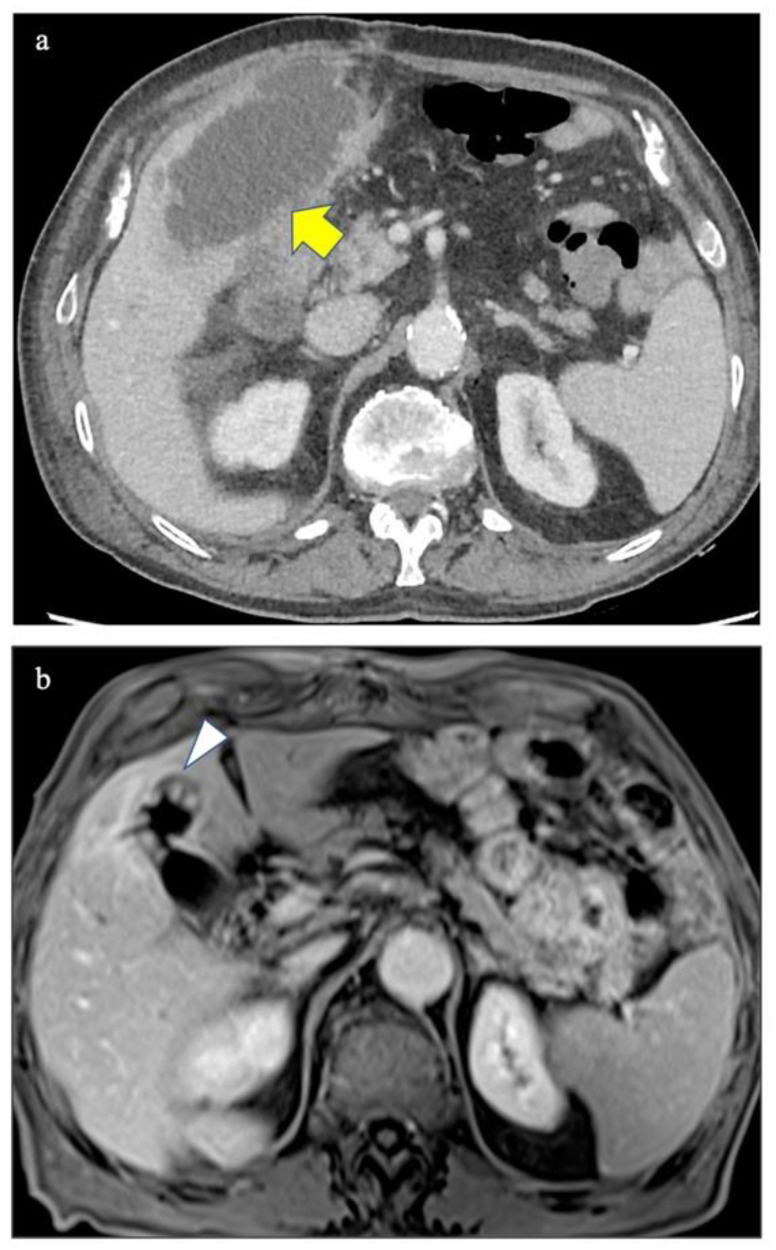
Axial contrast-enhanced Computed Tomography image of the upper abdomen well-demonstrating the acute cholecystitis with localized perforation (yellow arrow in (**a**)) and fat-saturated Volume Interpolated Breath-Hold Examination (VIBE) Magnetic Resonance image after contrast injection performed 90 days later showing multiple small pseudopolyps in the fundus of the gallbladder (white arrow in (**b**)).

**Figure 2:**
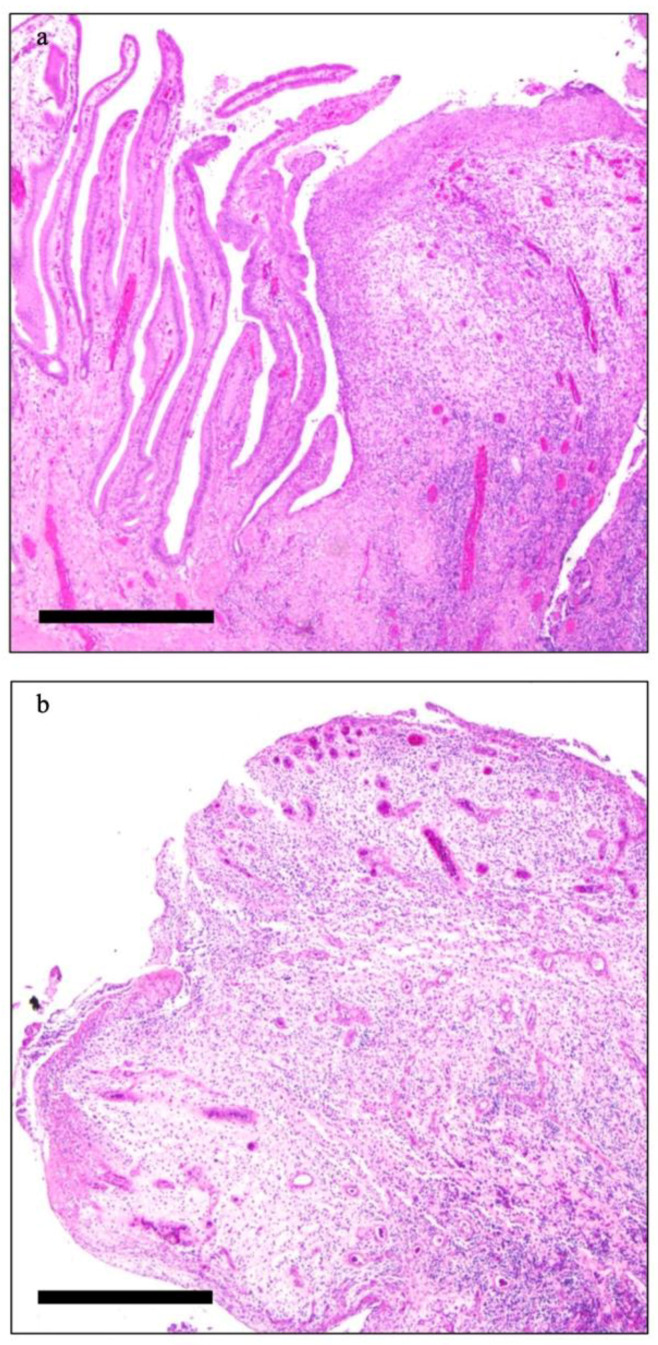
Histological image demonstrating papillary hyperplasia and an inflammatory pseudopolyp (**a**) and particular representative picture of one of the described inflammatory pseudopolyps observed in the gallbladder showing an ulcerated sessile mucosal projection characterized by edematous vascularized stroma with mixed type inflammatory infiltration (**b**) (scale bar 500 um).

**Table 1:** Summary of the records collected by a brief Pubmed literature search using the keywords “(acute acalculous cholecystitis) AND (coronary artery disease)”.

Publication Year	First Author	Title	Study Type	Number of Cases	Main Findings Related to AAC and Coronary Artery Disease
1986	Welling, R.E., et al. [12]	Gastrointestinal complications after cardiac surgery	Original Article	18 patients out of 1596 who underwent coronary artery bypass or valve replacement. had gastrointestinal complications	One patient underwent cholecystectomy for AAC
1988	Varma, D.G., et al. [13]	Computed tomography of gangrenous acute postoperative acalculous cholecystitis	Case report	1	Gangrenous AAC after two-vessel coronary artery bypass surgery
1989	Berger, H. et al. [14]	Percutaneous cholecystostomy in acute acalculous cholecystitis	Original Article	8	PC was successful in all patients with AAC. One patient had in anamnesis a coronary bypass operation
1993	Teranishi, K., et al. ^#^ [15]	A case of acute hemorrhagic gangrenous acalculous cholecystitis with bile peritonitis during anti-coagulant therapy after coronary-artery bypass grafting	Case report	1	A case of acute hemorrhagic, gangrenous acalculous cholecystitis after coronary-artery bypass grafting. Post-operative stasis of bile, swelling of the gallbladder, hypotension during cardiopulmonary bypass, and post-operative anti-coagulant therapy administered after open heart surgery have been proposed as etiological factors
1997	Saito, A., et al. [16]	Acute acalculous cholecystitis after cardiovascular surgery	Original Article	6	Examination of six cases of AAC after cardiovascular surgery and AAC. The authors suggest that post-surgical hypoperfusion of the gallbladder due to various factors may be the cause
1999	Fujiii, H., et al. [17]	Acute acalculous cholecystitis complicated by penetration into the liver after coronary artery bypass grafting	Case report	1	AAC with penetration into the liver in a 71-year-old woman 21 days after coronary artery bypass grafting. At histology, partial obstruction of the cystic artery due to atherosclerosis was found
2003	Funabiki, K., et al. [18]	Cholesterol crystal embolization (CCE) after cardiac catheterization: a case report and a review of 36 cases in the Japanese literature	Case Report	1	A 67-year old man developed AAC 12 days after coronary angiography which followed a previous coronary artery bypass grafting
2012	Chen, C.J., et al. [19]	Sonographic gallbladder abnormality is associated with intravenous immunoglobulin resistance in Kawasaki disease	Original Article	93 children with Kawasaki Disease	Five children with KD out of 11 with pathologic findings at abdominal ultrasound had AAC. Overall pathologic findings at US in children with KD seem to be associated with high levels of C-reactive protein, Glutamic-Pyruvic Transaminase, neutrophils and intravenous immunoglobulin resistance
2012	Van Stejin, J.H.M., et al. ^#^ [20]	Acute acalculous cholecystitis: not only in the intensive care department	Case Reports	2	Two patients with AAC are reported; one of them admitted to the coronary unit because of atherosclerotic vascular disease then died of sepsis
2014	Yi, D., et al. [21]	Hepatobiliary risk factors for clinical outcome of Kawasaki disease in children	Original Article	24 out of 67 children with KD had AAC	Coronary artery abnormalities were more frequent in patients with AAC
2019	Kang, W.D., et al. [22]	Clinical aspects of splenomegaly as a possible predictive factor of coronary artery changes in Kawasaki disease	Original Article	77 out of 396 examined patients underwent abdominal ultrasound	There were no cases of AAC at ultrasound among all investigated patients
2019	Lipe, D.N., et al. [23]	Kawasaki Disease Presenting as Acute Acalculous Cholecystitis	Case report	1	Eight-year-old boy affected by KD and with AAC
2021	Chen, B.Q., et al. [24]	Percutaneous cholecystostomy as a definitive treatment for moderate and severe acute acalculous cholecystitis: a retrospective observational study	Original Article	44	In patients with moderate to severe AAC who underwent PC, coronary heart disease or congestive heart failure are independent risk factors for relapse

PC = percutaneous cholecystostomy; AAC = acute acalculous cholecystitis; KD = Kawasaki Disease; ^#^ information extracted from the abstract only since the full-text was not in English.
